# Impact of Lifestyle Changes on Body Weight Gain During Nationwide Lockdown Due to COVID-19 Pandemic

**DOI:** 10.3390/jcm14072242

**Published:** 2025-03-25

**Authors:** Chisa Nishida, Hiroyuki Honda, Yuki Otsuka, Hideharu Hagiya, Yasuhiro Nakano, Kohei Oguni, Kazuki Tokumasu, Yasue Sakurada, Mikako Obika, Fumio Otsuka

**Affiliations:** Department of General Medicine, Okayama University Graduate School of Medicine, Dentistry and Pharmaceutical Sciences, 2-5-1 Shikata-cho, Kitaku, Okayama 700-8558, Japan; pfna5tx0@s.okayama-u.ac.jp (C.N.); otsuka@s.okayama-u.ac.jp (Y.O.); hagiya@okayama-u.ac.jp (H.H.); y-nakano@okayama-u.ac.jp (Y.N.); oguni-gim@okayama-u.ac.jp (K.O.); tokumasu@okayama-u.ac.jp (K.T.); pzaf6h9w@s.okayama-u.ac.jp (Y.S.); obika-m@cc.okayama-u.ac.jp (M.O.); fumiotsu@md.okayama-u.ac.jp (F.O.)

**Keywords:** COVID-19 pandemic, lockdown, weight gain, medical check-ups, lifestyle

## Abstract

**Background:** During the coronavirus disease 2019 (COVID-19) pandemic, people in Japan were urged to stay at home as much as possible, and this resulted in significant changes in lifestyle behavior. The new lifestyle included factors affecting both energy intake and energy consumption, and it is now thought that weight gain during the lockdown was the result of complex effects. The aim of this study was to determine the relationships among lifestyle habits, laboratory data, and body weight gain during the lockdown using medical check-up data. **Methods:** A total of 3789 individuals who had undergone consecutive medical check-ups during the period from 2018 to 2020 were included in this study. Participants whose body weight had increased by 5% or more were divided into two groups: a before-lockdown group (participants who had gained weight between 2018 and 2019) and an after-lockdown group (participants who had gained weight between 2019 and 2020). Physical measurements, laboratory data, and answers to six questions about lifestyle habits, for which information was obtained from the records from medical check-ups, were compared in the two groups. **Results:** There was no significant difference between the distribution of weight changes in 2018–2019 before the lockdown and the distribution of weight changes in 2019–2020 after the lockdown. The before-lockdown and after-lockdown groups both included about 7% of the total participants (279 and 273 participants, respectively). Diastolic blood pressure and levels of AST, ALT, and LDL-C were significantly higher in the after-lockdown group than in the before-lockdown group. The percentages of participants with alcohol consumption and exercise habits were significantly higher in the after-lockdown group than in the before-lockdown group, and an analysis by gender showed that the differences were significant for women but not for men. **Conclusions:** The distributions of weight changes before and during the COVID-19 pandemic were similar. Exercise habits and alcohol consumption might have been unique factors causing weight gain during the COVID-19 pandemic, particularly in women. Our findings suggest that the impact of behavioral restrictions and lifestyle changes during a pandemic may be different in men and women.

## 1. Introduction

Coronavirus disease 2019 (COVID-19) became a global pandemic after 11 March 2020, and many countries implemented lockdowns with bans on domestic and international travel in order to prevent the spread of infection. On 5 May 2023, more than three years after the outbreak, the pandemic was declared over. However, efforts are still being made in many countries to find ways to manage COVID-19 on a long-term and continuous basis. In Japan, a state of emergency was first declared on 7 April 2020, at the beginning of the pandemic, and people were asked to refrain from going out for non-essential reasons until 25 May 2020. Changes in eating habits and a decrease in physical activity during lockdowns were reported in many countries [1,2].

Excessive energy intake from food is known to be associated with obesity [3,4,5], while increased physical activity is known to be negatively correlated with weight gain [3,6,7]. Concerns about weight gain during the lockdown, known as ‘COVID-19 weight gain’, were reported in the Japanese media. While many negative consequences of a lockdown, such as unhealthy dietary habits and physical inactivity, were reported, several positive consequences for dietary habits and physical activity were also reported [2]. These positive consequences included an increase in fresh, homemade, and balanced meals [8], diets containing a wider range of foods [9], and a decrease in fast food consumption [10]. There was also an increase in physical activity during lockdowns due to increased use of a public bike-sharing system for commuting to work and for use on weekends [11] and maintenance of physical activity at home [12,13]. The new lifestyle associated with a lockdown includes factors affecting both energy intake and energy consumption, and it is now thought that weight gain during a lockdown is the result of complex effects. According to the National Health and Nutrition Survey of Japan, the average BMI of the Japanese population is approximately 23, and the proportion of people with obesity is 4.5%. Compared to other countries, Japanese people have a smaller body size and a lower baseline BMI, so even a small weight gain can have a significant impact.

Therefore, we conducted the present study to clarify the relationships among lifestyle habits, laboratory data, and body weight gain using medical check-up data for participants who had undergone medical check-ups for three consecutive years since 2018. We analyzed the relationships between body weight gain and lifestyle habits. Our findings will be useful for establishing approaches to provide health guidance during lockdowns.

## 2. Methods

### 2.1. Research Design and Participants

We conducted a retrospective observational study using secondary clinical data from a primary survey: medical check-up data from eight community hospitals in Okayama Prefecture and Kagawa Prefecture in Japan, including Marugame Medical Center, Kosei General Hospital, Tamano City Hospital, Kasaoka City Hospital, Okayama Kyokuto Hospital, Kurashiki Medical Center, Junpukai Health Maintenance Center, and Bizen City Hospital.

The study period was from 1 May to 31 June 2020, and the participants were individuals who had undergone medical check-ups for three consecutive years since 2018. The study period included the period of the first nationwide lockdown for COVID-19 in Japan from 16 April to 25 May 2020, a period that may have had effects on the participants’ lifestyles.

### 2.2. Data Processing and Definition of Weight Gain

Data from 3789 participants who underwent medical check-ups during the above-stated period were analyzed. In a recent study, body weight gain was defined as an increase in body weight of 5% or more compared to the previous year, because a 3–5% weight loss is generally set as a treatment target in health guidance for lifestyle-related diseases in Japan. A previous study on weight change and metabolic markers in Japanese people showed that a weight gain of more than 3 kg is associated with increased metabolic markers, such as triglycerides and blood pressure. A 3 kg increase in body weight corresponds to a weight gain of about 5% in the average Japanese body size [14]. The before-lockdown group in the present study included participants whose body weight had increased by 5% or more between 2018 and 2019, before the COVID-19 pandemic, and the after-lockdown group included participants whose body weight had increased by 5% or more between 2019 and 2020, which included the period of the COVID-19 lockdown.

### 2.3. Evaluation Contents

Age, sex, height, weight, body mass index (BMI), waist circumference, blood pressure, pulse rate, laboratory data, and answers to six questions about lifestyle habits were compared in the two groups. The laboratory data included data for lipids (low-density lipoprotein cholesterol (LDL-C), high-density lipoprotein cholesterol (HDL-C), and triglycerides (TG)), liver enzymes (aspartate aminotransferase (AST), alanine aminotransferase (ALT), and γ-glutamyl transpeptidase (γGTP)), levels of renal function markers (blood urea nitrogen (BUN) and creatinine (Cre)), and fasting plasma glucose (FPG) and hemoglobin A1c (HbA1c) levels. The six questions about lifestyle habits were included in the standard questionnaire for a medical check-up by the Ministry of Health, Labor, and Welfare. The questionnaire is used for individualized health guidance according to lifestyle. The questionnaire included questions about exercise habits, physical activity, snacking, amount of alcohol consumed per day, frequency of alcohol consumption, and motivation to improve lifestyle habits (Table 1). Data from 2019 for the before-lockdown group were compared with data from 2020 for the after-lockdown group.

### 2.4. Statistical Analysis

Statistical analysis was performed using Stata/SE 18.0 (StataCorp, 4905 Lakeway Dr, College Station, TX 77845, USA). The data were analyzed using the Mann–Whitney U test for continuous variables and ordinal scales, and Fisher’s exact test was performed for categorical variables. Statistical significance was set at a *p*-value < 0.05.

### 2.5. Ethical Considerations

Information about enrollment in the study was disclosed on each hospital’s website and by poster notice at each hospital, with an opportunity for a denial of participation, and we provided a contact point for the participants to opt out. The need for informed consent from each participant was waived. The study protocol was approved by the Ethical Committee of Okayama University Hospital (No. 2211-021) and adhered to the tenets of the Declaration of Helsinki and the Ethical Guidelines for Medical and Health Research Involving Human Subjects.

## 3. Results

**Figure 1:**
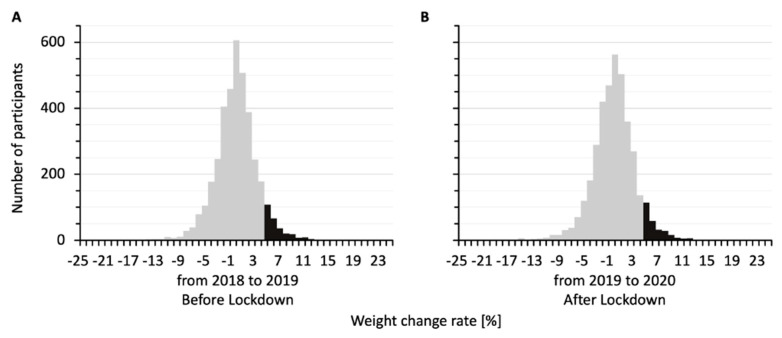
Distributions of percent weight changes for the study participants from 2018 to 2019 (**A**) and from 2019 to 2020 (**B**). The bars show similar distributions, with 279 participants gaining 5% or more in weight from 2018 to 2019 and 273 participants gaining 5% or more in weight from 2019 to 2020 (black bars).

Figure 1 shows the distributions of percent weight changes for the 3789 participants from 2018 to 2019, before the lockdown (Figure 1A), and from 2019 to 2020, after the lockdown (Figure 1B). The two histograms were similarly distributed. The percent weight changes were 0.44 ± 0.06% from 2018 to 2019 and 0.27 ± 0.06% from 2019 to 2020. In both the before-lockdown and after-lockdown groups, participants who gained 5% or more in weight accounted for about 7% of the total participants (279/3789 and 273/3789 participants, respectively) in each histogram.

Table 2 shows a comparison of the backgrounds of the participants in the before-lockdown and after-lockdown groups. The median ages [interquartile range] of the participants were 46 [40, 52] years in the before-lockdown group and 48 [42, 54] years in the after-lockdown group. Diastolic blood pressure was significantly higher in the after-lockdown group than in the before-lockdown group (74 [65, 84] vs. 72 [63, 80]; *p* < 0.05).

**Figure 2:**
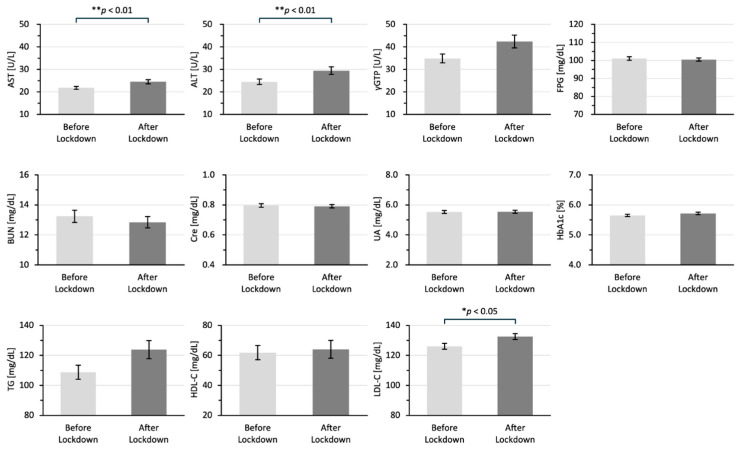
Differences in laboratory data for the before-lockdown and after-lockdown groups. All of the laboratory data are shown as means ± SEM in 2019 for the before-lockdown group and in 2020 for the after-lockdown group. The data were analyzed by using the Mann–Whitney U test; * *p* < 0.05 and ** *p* < 0.01 indicate statistically significant differences. AST: aspartate aminotransferase, ALT: alanine aminotransferase, γGTP: γ-glutamyl transpeptidase, BUN: blood urea nitrogen, CRE: creatinine, UA: uric acid, TG: triglycerides, HDL-C: high-density lipoprotein cholesterol, LDL-C: low-density lipoprotein cholesterol.

Figure 2 shows the differences between the laboratory data in the two groups. All of the laboratory data are shown as means ± standard error of the mean. The laboratory data for AST (25 ± 0.95 vs. 22 ± 0.67 U/L; *p* < 0.001), ALT (29 ± 1.69 vs. 25 ± 1.20 U/L; *p* < 0.01), and LDL-C 133 ± 2.01 vs. 126 ± 1.99 mg/dL; *p* < 0.05) in the after-lockdown group were significantly higher than those in the before-lockdown group.

**Figure 3:**
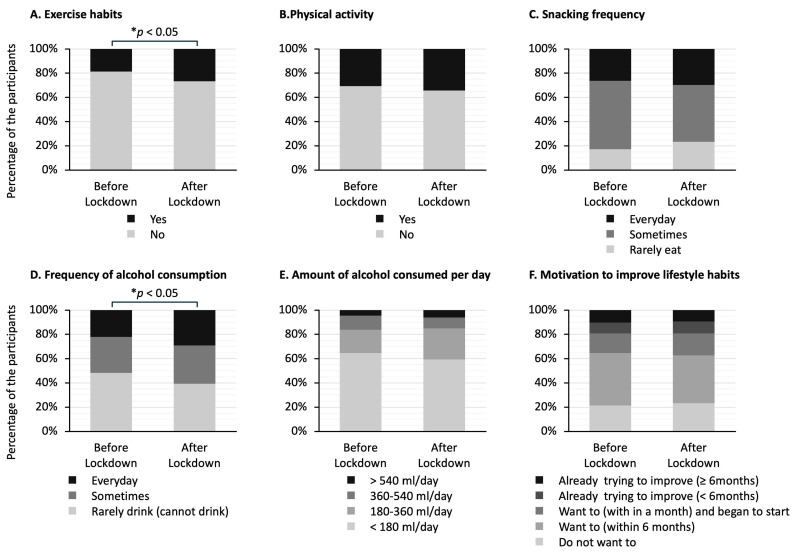
Differences in answers to the questionnaire about lifestyle habits in the before-lockdown and after-lockdown groups. The stacked bar graphs show the percentage of answers to each question. The question regarding exercise habits asks whether the participants have been engaging in light sweaty exercise for at least 30 min at a time, at least 2 days a week, for at least 1 year. The question regarding physical activity asks whether the participants perform walking or equivalent physical activity in daily life for at least one hour per day. The question regarding snacking frequency asks whether the participants consume snacks or sweet beverages between meals. The question regarding frequency of alcohol consumption asks how often the participants drink sake, shochu, beer, wine, whisky, brandy, etc. The question regarding the amount of alcohol consumed per day asks how much the participants drink sake (180 mL), beer (500 mL), shochu 25% (110 mL), double whisky (60 mL), or two glasses of wine (240 mL) per day. The question regarding motivation to improve lifestyle habits asks whether the participants want to improve their life habits of eating and exercising. The data in (**A**,**B**) were analyzed using Fisher’s exact test, and the data in (**C**–**F**) were analyzed using the Mann–Whitney U test; * *p* < 0.05 indicates statistically significant differences.

Figure 3 shows the answers to questionnaires about lifestyle habits in the two groups. The stacked bar graphs show the percentage of responses to each question. Of the six answers to questions, the ones that showed significant differences between the two groups were exercise habits (Figure 3A) and frequency of alcohol consumption (Figure 3D). The proportion of participants who answered that they had exercise habits was higher in the after-lockdown group than in the before-lockdown group (27% vs. 19%; *p* < 0.05) (Figure 3A). The proportion of participants who answered that they drank alcohol at least occasionally was significantly higher in the after-lockdown group than in the before-lockdown group (61% vs. 52%; *p* < 0.05) (Figure 3D).

**Figure 4:**
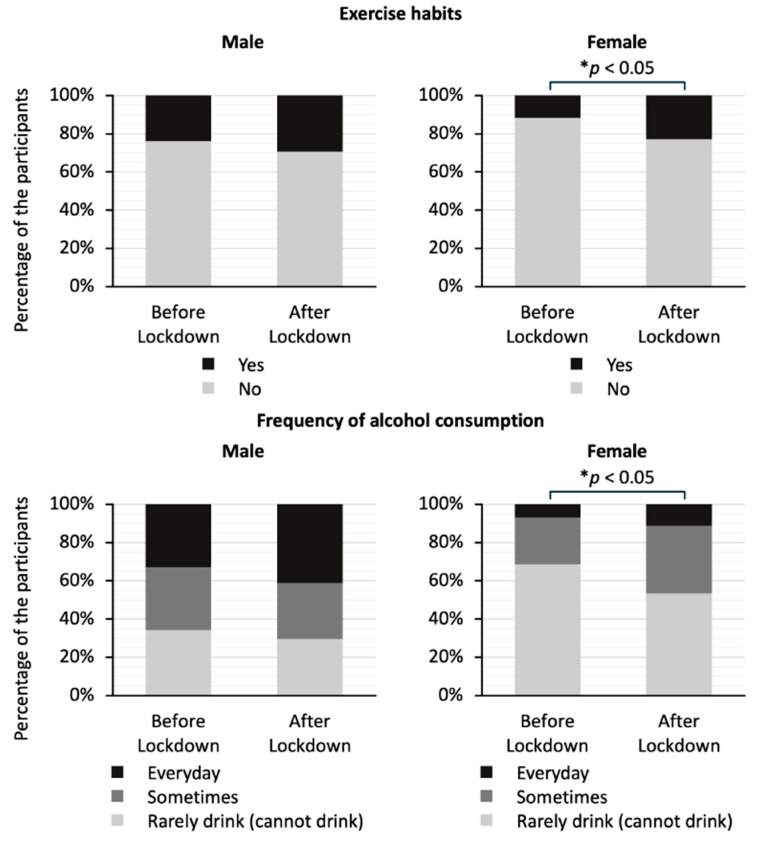
Comparison of answers to the questionnaire about frequency of alcohol consumption and exercise habits by gender between the before-lockdown and after-lockdown groups. The stacked bar graphs show the percentage of responses to each question. The question regarding exercise habits asks whether the study participants have been engaging in light sweaty exercise for at least 30 min at a time, at least 2 days a week, for at least 1 year. The question regarding frequency of alcohol consumption asks how often the participants drink sake, shochu, beer, wine, whisky, brandy, etc. The data on exercise habits were analyzed using Fisher’s exact test, and the data on frequency of alcohol consumption were analyzed using the Mann–Whitney U test; * *p* < 0.05 indicates statistically significant differences.

Figure 4 shows a comparison of exercise habits and drinking frequency by gender in the two groups. The stacked bar graphs show the percentage of responses to each question. There was no significant difference in the exercise habits or drinking frequency for men. On the other hand, the proportion of women who answered that they had exercise habits was significantly higher in the after-lockdown group than in the before-lockdown group (23% vs. 12 %; *p* < 0.05). In addition, the proportion of women who answered that they drank alcohol at least occasionally was significantly higher in the after-lockdown group than in the before-lockdown group (46% vs. 31%; *p* < 0.05).

## 4. Discussion

In the present study, we found that there was no difference in the distributions of weight changes for the participants before and during the COVID-19 pandemic. Focusing on participants whose body weight increased by 5% or more, diastolic blood pressure and levels of AST, ALT, and LDL-C were significantly higher in the participants in the after-lockdown group than in the participants in the before-lockdown group. The percentages of participants with alcohol consumption and exercise habits were significantly higher in the after-lockdown group than in the before-lockdown group, and the differences were particularly notable for women.

During the COVID-19 lockdown in Japan, the Japanese media reported the impact of the lockdown on eating and exercise habits and the associated ‘COVID-19 weight gain’. The impact of the COVID-19 lockdown on lifestyle and the resulting weight changes were also reported worldwide. Reports of changes in body weight during the COVID-19 lockdown have been varied, with some reports of weight gain and other reports of weight loss, even in the same country [15,16]. There have been reports on changes in body composition, such as reduced free fat mass, without any change in body weight before and during the COVID-19 pandemic [17]. There have also been reports of weight loss due to malnutrition, which is particularly worrying in the elderly [18]. To date, no definite conclusions regarding weight changes during the COVID-19 lockdown have been reached. In the present study, the mean percent weight change for all the participants from 2019 to 2020, during the COVID-19 pandemic, was 0.3%, which was not significantly different from the mean percent weight change of 0.4% from 2018 to 2019, before the COVID-19 pandemic. For the young generation, during the peri-COVID-19 lockdown periods, we previously showed, through an analysis of a national database, that the prevalence of childhood obesity in Japan had significantly increased and that there were sharp increases in the prevalences of obesity in junior high school boys and elementary and junior high school girls starting from the late 2010s. Dietary changes and decreased exercise time, as well as the lockdown during the COVID-19 pandemic might have contributed to the increased prevalence of obesity in the young generation [19].

Weight gain and obesity are known to cause metabolic disorders, including high blood pressure [20], lipid abnormalities [21], and steatotic liver disease [22]. It has been reported that changes in systolic and diastolic blood pressure are associated with changes in body weight and that changes in diastolic blood pressure are associated with physical activity independently of changes in body weight [23]. Increased BMI is associated with decreased HDL-C and increased LDL-C and TG, but the relationships of HDL-C and TG with BMI are stronger [21]. In the present study, diastolic blood pressure, transaminases, and LDL-C were significantly higher in the after-lockdown group than in the before-lockdown group, although the participants in both groups gained more than 5% body weight. All of these results were within normal ranges and all of the differences were numerically very small and not considered clinically significant. Previous studies on metabolism during the COVID-19 pandemic showed negative effects on bone mineral density with no change in body fat mass, which may have been influenced by lifestyle [24]. There were no differences between the two groups in body weight, BMI, and waist circumference, suggesting that the differences in physical measurements and laboratory data between the two groups may have been caused by lifestyle changes other than weight gain and obesity.

It is known that physical activity is closely related to weight change [25]. Since physical activity affects energy expenditure, there is a negative correlation between the amount of physical activity and weight change [26]. Reduced physical activity is one of the triggers for obesity, and it has been reported that reduced physical activity during a lockdown is associated with weight gain and obesity [27,28,29,30]. In the present study, the percentage of participants with an exercise habit was significantly higher in the after-lockdown group than in the before-lockdown group, and this result seems to contradict the results of previous studies mentioned above. On the other hand, it was reported that there was an increase in moderate exercise during the COVID-19 pandemic compared to that before the pandemic among employees participating in health promotion programs [31]. Many of the participants in the present study had received health check-ups at work and were in relatively good health; they also received annual health check-ups during the COVID-19 epidemic, suggesting that they may have had a high level of awareness of health and exercise.

In this study, the percentage of participants with an exercise habit was significantly higher in the after-lockdown group than in the before-lockdown group, especially among women. Common reasons for participating in a sport and physical activity include weight management, social interaction, and enjoyment [32]. Older people are more motivated by the prevention of health problems and social interaction than younger people [33,34]. Both men and women tend to prefer exercise that is close to home, does not cost a lot of money, and can be done alone [35]. Among women, maintaining weight and shape and improving appearance were reported to be motivations for exercise [32,35]. It has also been reported that women prefer to be active at set times and are less likely to be outdoors [35]. During the lockdown, women were more likely to have private time and had enough time to improve their appearance, which may have motivated them to exercise more.

This study reveals that participants in the after-lockdown group consumed alcohol more frequently than participants in the before-lockdown group. A decrease in the frequency of eating out may have led to a decrease in alcohol consumption. Restrictions on eating out varied from country to country, and in countries in which restaurants were closed but the sale of alcohol for out-of-store consumption was not banned, there was concern about increased drinking at home [36]. During the lockdown in Japan, restaurants were asked by the government to reduce their opening hours. According to the Family Income and Expenditure Survey 2020 of the Statistics Bureau of Japan, alcohol consumption expenditure in restaurants in April and May in 2020 decreased by 89.1% year-on-year, while expenditure for alcohol consumption at home increased by 24.7% year-on-year. Convenience and cost were reported as reasons for choosing to drink at home [37]. The decline in eating out due to the lockdown may have led to a shift from drinking at restaurants to drinking at home, the latter of which is cheaper and easier than eating out and may have led to increased drinking frequency. In our earlier study on long COVID, we showed that patients with long COVID who had higher glucose levels and also had higher BMI, liver dysfunction, and dyslipidemia had a higher rate of drinking alcohol and required longer periods for recovery from long COVID, suggesting that lifestyle-related issues, including drinking habits, could also be involved in post-COVID-19 health conditions [38].

In this study, women in the after-lockdown group consumed alcohol more frequently than women in the before-lockdown group. The finding in the present study of increased frequency of alcohol consumption among women during the COVID-19 lockdown is consistent with findings in studies in other countries [39,40,41]. It has been reported that women are more likely than men to buy alcohol in supermarkets [37]. Women tend to drink to relax and take a temporary break from work and household responsibilities [42]. Mothers with children tend to choose to drink at home because of the hassle and expense of hiring a babysitter for outside drinking [42]. During the lockdown, women’s childcare and housework burdens were greater than ever before [43,44,45]. According to the Organization for Economic Co-operation and Development’s (OECD) economic survey, Japanese men spent an average of 41 min per day on housework, one-third less than the OECD average, while women spent about three hours per day more on housework than men, suggesting that women spent more time undertaking these roles during the pandemic. In addition, more women than men lost their jobs during the lockdown, as women’s employment is generally more precarious [43]. The COVID-19 pandemic had a greater impact on women than on men, both in terms of work and private life. Studies on the psychological impact of the COVID-19 pandemic [46] revealed greater psychological stress in women than in men. Women’s drinking behavior is known to be closely related to psychological stress [47]. The increased frequency of drinking among women in the present study may be the result of coping behavior for a high level of psychological stress during confinement.

## 5. Limitations

This study has several limitations. First, many of the participants in this study underwent medical check-ups at work and were healthy enough to work. They may have had high health literacy because they underwent annual medical check-ups even during the COVID-19 pandemic. Second, the presence of lifestyle diseases and their treatment status were unknown, and the possibility of other confounding factors, such as dietary habits and sleeping hours, which are generally associated with obesity, were not examined. Exercise volume affects appetite, as well as body weight, with moderate-intensity exercise increasing appetite [48]. Considering the increase in exercise habits among the participants gaining 5% or more in weight, data on exercise intensity and food intake are needed. Based on the statistics regarding frequency of alcohol consumption by gender between the before-lockdown and after-lockdown groups, the power is 58%, and larger sample sizes are needed to conclude the findings of this study. Third, the medical check-up questionnaire used in this study was self-administered, and the participants’ awareness may have influenced the results. It has been reported that individuals who had exercise habits before the pandemic tended to exercise more during the pandemic [49,50]. Since the question about exercise habits asked whether the participants had been exercising for more than one year, it is possible that the participants may have been exercising before the COVID-19 pandemic and that this may not fully reflect changes in exercise habits during the pandemic.

## 6. Conclusions

In summary, we showed that the distributions of weight changes before and during the COVID-19 pandemic were similar. Focusing on participants who gained 5% or more in weight, the present study uncovers that frequent drinking and exercise habits affected weight gain during the COVID-19 pandemic, particularly among women. The difference between men and women found in this study suggests that the impact of behavioral restrictions and lifestyle changes associated with epidemics of emerging infectious diseases on health status may differ by gender. It may be necessary to consider a gender-conscious approach when providing health guidance during lockdowns.

## Figures and Tables

**Table 1:** Standard questionnaire for medical check-up in Japan.

Questions	Answers
A. Exercise habits	
Have you been in the habit of exercising to sweat lightly for over 30 min a time, 2 times weekly, for over a year?	1. Yes 2. No
B. Physical activity	
In your daily life, do you walk or do any equivalent amount of physical activity for more than one hour a day?	1. Yes 2. No
C. Snacking frequency	
Do you eat snacks or drink sweet beverages between meals?	1. Everyday 2. Sometimes 3. Rarely eat
D. Frequency of alcohol consumption	
How often do you drink? (Sake, shochu, beer, wine, whisky, brandy, etc.)	1. Everyday 2. Sometimes 3. Rarely eat
E. Amount of alcohol consumed per day	
How much do you drink per day? Sake (180 mL), beer (500 mL), shochu 25% (110 mL), double whisky (60 mL), two glasses of wine (240 mL)	1. Less than 180 mL 2. 180–360 mL 3. 360–540 mL 4. More than 540 mL
F. Motivation to improve lifestyle habits	
Do you want to improve your life habits of eating and exercising?	1. Do not want to 2. Want to (within 6 months) 3. Want to improve in near future (within a month) and began to change 4. Already trying to improve (less than 6 months) 5. Already trying to improve (over 6 months)

**Table 2:** Comparison of backgrounds of participants in the before-lockdown and after-lockdown groups.

	Before-Lockdown Group	After-Lockdown Group	*p* Value
	(*n* = 279)	(*n* = 273)	
Patients’ profiles			
Age [years]	46 [40, 52]	48 [42, 54]	
Gender [male % (*n*)/female % (*n*)]	55.6 (155)/44.4 (124)	57.5 (157)/42.5 (116)	0.643
Height [cm]	165 [158, 172]	166 [160, 171]	0.954
Weight [kg]	65 [56, 72]	65 [57, 74]	0.399
BMI	24 [21, 26]	24 [22, 26]	0.351
Waist circumference [cm]	83 [77, 89]	84 [77, 89]	0.648
SBP [mmHg]	119 [107, 128]	119 [110, 132]	0.076
DBP [mmHg]	72 [63, 80]	74 [65, 84]	* <0.05
PR [/min]	70 [64, 75]	68 [63, 74]	0.263

Data are shown as medians [IQR: interquartile ranges] and numbers (%) and were analyzed using the Mann–Whitney U test or Fisher’s exact test; * *p* < 0.05 indicate statistically significant differences. BMI: body mass index; SBP: systolic blood pressure; DBP: diastolic blood pressure; PR: pulse rate.

## Data Availability

Detailed data will be provided upon request to the corresponding author.

## Abbreviations

Alanine aminotransferase (ALT); aspartate aminotransferase (AST); body mass index (BMI); blood urea nitrogen (BUN); coronavirus disease 2019 (COVID-19); creatinine (Cre); fasting plasma glucose (FPG); γ-glutamyl transpeptidase (γGTP); hemoglobin A1c (HbA1c); high-density lipoprotein cholesterol (HDL-C); low-density lipoprotein cholesterol (LDL-C); Organization for Economic Co-operation and Development (OECD); triglycerides (TG).
